# Calciphylaxis in a patient with hypoparathyroidism and MEN-1 syndrome

**DOI:** 10.1530/EDM-23-0009

**Published:** 2023-12-08

**Authors:** Isabelle van Heeswijk, Antonia Ugur, Lynsey Havill, Rebecca Kinton, David Hughes

**Affiliations:** 1 Department of Endocrinology, University Hospitals Derby & Burton NHS Trust, Derby, UK

**Keywords:** Adult, Male, White, United Kingdom, Parathyroid, Diabetes, Thyroid, MEN1, Unique/unexpected symptoms or presentations of a disease, December, 2023

## Abstract

**Summary:**

Calciphylaxis is a rare disorder characterised by the development of painful necrotic skin lesions. Occlusion of cutaneous arterioles due to ectopic calcification leads to potentially life-threatening widespread skin loss. Most cases occur in patients with chronic renal disease, which leads to dysregulation of calcium and phosphate homeostasis. Only a handful of case reports exist describing calciphylaxis occurring in patients without chronic renal disease but with hypoparathyroidism. We report on a unique case of a 53-year-old man with multiple endocrine neoplasia type 1 syndrome and acquired hypoparathyroidism due to total parathyroidectomy who went on to develop calciphylaxis following cardiac surgery.

**Learning points:**

- Calciphylaxis most commonly occurs in the context of chronic renal disease but can rarely occur in its absence as a consequence of calcium and phosphate dysregulation.
- Patients who develop necrotic skin lesions in the presence of hypoparathyroidism require an urgent dermatological opinion.
- Mortality from calciphylaxis is high, with the majority of deaths occurring secondary to sepsis.
- Management of calciphylaxis requires a multidisciplinary team approach to manage wound healing, infections and pain.
- Recovery with full rehabilitation from calciphylaxis can take months to years.

## Background

Calciphylaxis is a rare disorder characterised by painful necrotic lesions of the skin and subcutaneous tissue. The underlying pathogenesis is extraosseous deposition of calcium crystals with microvascular calcification, resulting in occlusion of cutaneous arterioles. An elevated calcium × phosphate product ((serum Ca) × (serum PO_4_)) that is greater than >5.6 mmol^2^/L^2^ has a specificity of 95% and sensitivity of 21% for calciphylaxis (1). It is recommended in end-stage renal patients to keep the calcium × phosphate product <4.4 mmol^2^/L^2^ in order to minimise the risk of developing calciphylaxis (2).

The condition is rare, with a reported incidence in Europe of 4 per 10 000 patients (3). Most cases occur in patients with end-stage renal failure due to failure to regulate serum phosphate and calcium levels (4). Only 10–20% cases occur in patients without significant renal disease. Other risk factors include hyperparathyroidism, warfarin therapy (5), diabetes and obesity. Only a handful of case reports describe the condition occurring in the context of hypoparathyroidism (6).

Due to its rarity, there is no evidence-based consensus on the management of calciphylaxis. Treatment usually involves (i) prevention and aggressive treatment of infections, (ii) supportive multidisciplinary wound care and (iii) maintenance of a normal serum calcium and phosphate level. One-year mortality from the condition is ~65%, with death usually occurring as a result of sepsis (1, 7).

## Case presentation

The case concerns a 53-year-old Caucasian gentleman with a past medical history of multiple endocrine neoplasia type 1 (MEN-1). He had previously undergone four-gland parathyroidectomy with forearm parathyroid tissue auto-transplantation in January 2020 to treat hypercalcaemia secondary to primary hyperparathyroidism. The auto-transplanted graft failed, leading to hypoparathyroidism. Parathyroid hormone (PTH) levels at diagnosis and immediately pre-operatively were 234 ng/L and 274 ng/L, respectively. Post-operatively, PTH levels were 5 ng/L and over the subsequent 3 years of monitoring have remained below the normal range. Calcium levels were maintained with alfacalcidol and calcium supplements.

His MEN-1 was confirmed on genetic testing, demonstrating a c.758C>Tp.(ser253leu) mutation in the *MEN-1* gene. He initially presented with a <1 cm non-secreting pancreatic neuroendocrine tumour confirmed histologically to have low proliferation indexes and monitored with annual radiological surveillance. A later finding of parathyroid-dependent hypercalcaemia led to investigation for MEN-1 syndrome. In addition, he had a congenital bicuspid aortic valve with an ascending thoracic aortic aneurysm. Consequently, he developed aortic stenosis, with regurgitation resulting in early-onset heart failure. In January 2022, a bioprosthetic aortic valve was inserted at a regional cardiothoracic centre. Post-operatively, he developed hospital-acquired pneumonia and a pre-renal acute kidney injury. Creatinine peaked at 204 on day 16 post-operatively. At that point, the development of ‘friction lesions’ on his buttocks, lower abdomen and lower limbs was described. Following recovery from pneumonia, he was repatriated to his local hospital for rehabilitation, where these lesions progressed to small areas of necrosis. Following an urgent dermatology consultation, the differential diagnosis of purpura fulminans, heparin-induced necrosis or calciphylaxis was made. A skin biopsy was performed in order to confirm the diagnosis.

## Investigation

Skin biopsy demonstrated numerous foci of epidermal and fat necrosis with widespread dermal oedema. Fibrinoid necrosis was evident in small vessels of the dermis and focally within the subcutis. Von Kossa staining (a histological stain used to demonstrate mineralisation) demonstrated focal calcification of vessel walls as well as calcific deposits within the subcutis. These findings were in keeping with the histological diagnosis of calciphylaxis.

Biochemical results at the time of skin biopsy were in keeping with the overtreatment of his hypoparathyroidism (see Table 1). Subsequent fluctuations in calcium and phosphate levels are summarised in Table 2.

**Table 1:** Baseline biochemistry results.

Tests	Reference range	Results
Adjusted calcium (mmol/L)	2.20–2.60	3.03 ↑
Calcium (mmol/L)	2.20–2.60	2.71 ↑
Phosphate (mmol/L)	0.8–1.5	1.88 ↑
Albumin (g/L)	35–50	14 ↓
Calcium × phosphate product (mmol^2^/L^2^)	<4.4	5.7 ↑
Parathyroid hormone (ng/L)	15–65	3 ↓
Sodium (mmol/L)	135–145	132 ↑
Potassium (mmol/L)	3.5–5.3	5.9 ↑
Urea (mmol/L)	2.5–7.8	23.5 ↑
Creatinine (µumol/L)	59–104	200 ↑
eGFR (mL/min/1.73m^2^)	>90	32 ↓

**Table 2:** Key events with corresponding medication and biochemistry results.

Weeks following diagnosis	Baseline	2 weeks	8 weeks	21 weeks	23 weeks	31 weeks	45 weeks
	At diagnosis	Following cessation of calcium supplements and alfacalcidol	Stabilisation of medication	Diagnosis of hypercalcaemia of immobility	Following treatment with bisphosphonate	Prior to attending football match	2 months following discharge and return of mobility
Adjusted calcium (mmol/L)	3.03	1.79	2.27	3.45	2.82	2.75	2.08
Phosphate (mmol/L)	1.88	3.42	1.4	1.41	1.51	1.05	1.42
Calcium × phosphate product (mmol^2^/L^2^)	5.7	6.12	3.2	4.86	4.26	2.89	2.95
Total daily oral dose of alfacalcidol, µg	1	0	0.6	0	0	0	0.5
Total daily oral dose of calcium supplement*, mg	3000	0	4750	0	0	0	500
Pamidronate infusion, mg				30^†^			

*Expressed as total weight of calcium salt; ^†^weekly once for 2 weeks.

## Treatment

On recognising the rarity, complexity and serious nature of the condition, the opinions of multiple disciplines were sought, including renal, endocrinology, cardiology, general surgery, orthopaedics, vascular surgery, palliative care team, acute pain team, dermatology, dietetics, tissue viability and plastic surgery.

His management plan throughout remained conservative, due to his complex comorbidities and frailty following cardiac surgery, at odds with his young age. Consequently, no surgical debridement was undertaken for the treatment of the calciphylaxis lesions in agreement with the tissue viability and plastic surgery teams.

**Figure 1:**
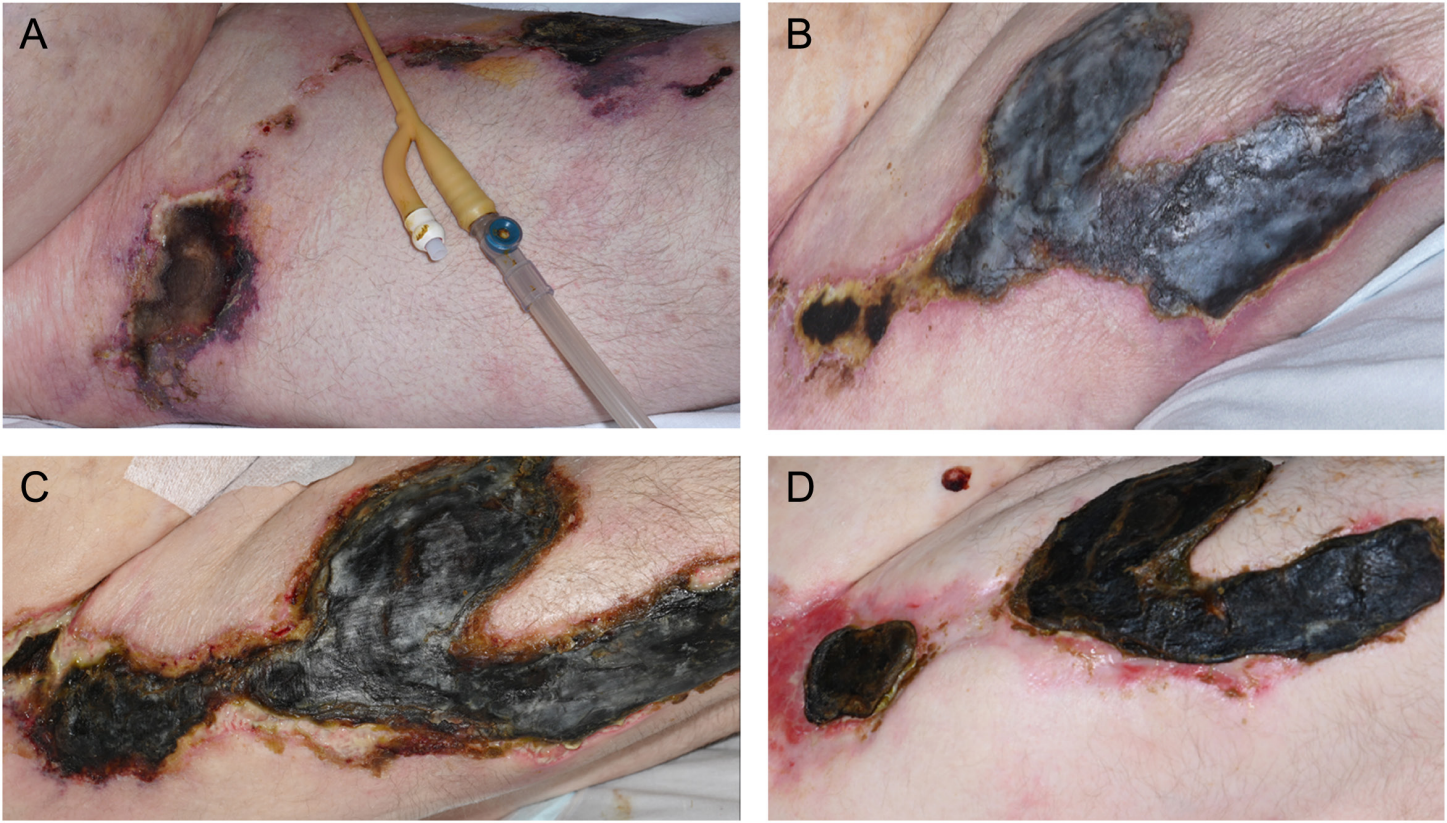
Right lateral thigh – (A) 2 weeks post diagnosis, (B) 1 month post diagnosis, (C) 3 months post diagnosis, and (D) 6 months post diagnosis.

Regular wound dressings were performed with intermittent photographic documentation of the size of the lesions (see Fig. 1A, B, C and D). An analgesia plan was formulated in consultation with the acute pain and palliative care teams to manage pain from the necrotic wounds, especially during dressing changes. Antibiotics were required frequently to manage systemic inflammatory response syndrome (SIRS) from opportunistic infections of his wounds. Life-threatening sepsis was prevented by early, aggressive antibiotic therapy with broad-spectrum agents such as intravenous piperacillin and tazobactam or meropenem. This was followed by appropriate microbiology culture-led stepdown. Subsequently, the microbiology team recommended antibiotic prophylaxis with 500 mg oral flucloxacillin twice daily. This reduced the frequency of intravenous broad-spectrum antibiotics and subjectively improved wound healing and general well-being.

Alfacalcidol and calcium supplements were titrated to reduce the calcium × phosphate product (see Table 2). The initial managing team responded to hypercalcaemia with cessation of alfacalcidol and calcium supplements, resulting in hyperphosphataemia and hypocalcaemia and an overall increase in calcium × phosphate product. On endocrinology advice, calcium acetate was initiated both to act as a phosphate binder and to treat symptomatic hypocalcaemia. This contrasts with the management of most cases of renal disease-related calciphylaxis, where non-calcium-containing phosphate binders are preferred. The dose was titrated to achieve calcium and phosphate levels within the normal range. The calcium-chelating agent sodium thiosulphate, which is often used in the treatment of renal-related calciphylaxis, was not administered, as it was felt that the risks of central venous cannulation in the context of recent cardiac surgery and recurrent sepsis outweighed any potential benefits of its use.

Although previously fit and well, the patient had become largely confined to bed, requiring hoisting for transfers due to the painful skin lesions. Around 3 months following diagnosis, the patient’s calcium levels began to spontaneously climb again, resulting in downtitration of both alfacalcidol and calcium supplements and ultimately their cessation. Despite cessation, his serum calcium levels continued to climb and remained elevated (see Table 2). Following exclusion of other causes of hypercalcaemia, he was diagnosed with hypercalcaemia secondary to immobility. As a result, two infusions of pamidronate disodium were administered to stabilise calcium levels.

Recovery was further complicated by intermittent episodes of haemorrhage from the wounds, requiring multiple blood transfusions and cessation of therapeutic anticoagulation for previous pulmonary embolus (PE).

In addition, extensive physiotherapy and occupational therapy were required to restore mobility. After being nursed in bed for more than 9 months, the therapy team successfully rehabilitated the patient to the point that he could attend a much-anticipated international football match being seated in a wheelchair. There followed a rapid improvement in his mobility, and he began to walk short distances with a Zimmer frame a week later.

## Outcome and follow-up

In total, the patient was hospitalised for 10 months with multiple medical complications following his cardiac surgery. Despite many setbacks, he survived to discharge. Significant wound healing was achieved with conservative management, with a reduction in the size of the lesions evident (see Fig. 1A, B, C and D). A combination of palliative care input and resolution of the wounds also resulted in significant improvements in his reported pain levels. He is under ongoing outpatient follow-up by the endocrinology team with regular biochemical monitoring. Although he was initially discharged without needing to continue alfacalcidol and calcium supplements, the recovery of his mobility eventually led to his calcium levels falling and thus their resumption. He is now walking and living independently at home with his family.

## Discussion

Although most often associated with renal disease, calciphylaxis can rarely occur in the context of hypoparathyroidism. Case reports describing calciphylaxis with hypoparathyroidism attribute the aetiology to complications of calcium management, with calciphylaxis commonly being triggered by treatment with vitamin D analogues or recombinant PTH (8). In this case, we suspect that calcium and phosphate levels rose due to post-operative acute kidney injury. The combination of reduced renal maintained homeostasis of calcium and phosphate with impaired excretion of alfacalcidol metabolites led to a raised calcium × phosphate product, which triggered extraosseous calcium deposition.

There are no evidence-based guidelines for the management of calciphylaxis in this scenario. In general, management focusses on the reduction of the calcium × phosphate product and supportive care whilst the necrotic wounds heal. Regular wound care, pain management and aggressive treatment of infections were the clinical cornerstone of our patient’s treatment. A multi-professional team approach ensured that we were able to utilise the skills, knowledge and experience from multiple disciplines to provide comprehensive care.

A meta-analysis of previously reported clinical interventions used for calciphylaxis – sodium thiosulfate, surgical parathyroidectomy, calcimimetics, hyperbaric oxygen therapy and bisphosphonates – did not demonstrate that any of these would provide a clinically significant benefit in this scenario (3). This meta-analysis and other case series also reflect on the high mortality from the condition (~65%).

In the case of our patient, only phosphate binders were used in the acute phase. The use of bisphosphonates occurred months later, with the primary indication to treat immobility hypercalcaemia rather than to directly manage the calciphylaxis. This is the first time hypercalcaemia secondary to immobility has been reported in a patient with hypoparathyroidism and MEN-1.

In addition to his medical and nursing care, we also believe that time, family support, faith and the motivation to attend a much-anticipated sporting event with his family also had a significant part to play. Ultimately, the combination of clinical care and a positive attitude was what enabled him to recover.

In conclusion, our case describes an often-fatal rare complication of hypoparathyroidism occurring in unusual circumstances where the management successfully led to the survival of the patient.

## Declaration of interest

There is no conflict of interest that could be perceived as prejudicing the impartiality of the study reported.

## Funding

This research did not receive any specific grant from any funding agency in the public, commercial or not-for-profit sector.

## Patient consent

Written informed consent for publication of their clinical details and/or clinical images was obtained from the patient.

## Patient’s perspective

I spent 1 year in hospital with a super rare condition called “Calciphylaxis”. It is dead skin eating away at your living skin. Excruciatingly painful. I was bed bound and couldn’t walk. Even with all the pain killers I was still in so much pain. It took nearly 3 months to diagnose me. They tried everything they could think of to heal it but nothing happened. Eventually once they had diagnosed me they finally found some dressings for my legs and sides. These new dressings didn’t stick to my skin like the other dressings did.

I died at least twice and thank God was brought back to life. One weekend I bled out. There was [sic] rivers of blood, we are talking about Biblical plagues level event, with the walls painted red with blood. I am not sure how much blood I lost but they gave me 5 pints of blood over that weekend. Finally the bleeding just stopped and afterwards I saw visible progress in my healing.

The hospital staff were wonderful. They would take care of me. All this time my wife was an absolute rock. She came to visit when she could. It hit my family really hard. Each day was lived in fear, ‘was this the day I would die’. My oldest daughter was scared to visit as I looked so “Skeletal”. I cried so much when she said that and I knew at that time just how hurt they all were.

I am now out of the hospital and in the final stages of my healing. I am learning to walk again. I was known and called “The Miracle Man” by nurses, doctors and medical staff. My family is so glad that I am home. The fear has gone and we’re are rebuilding our lives and family again.
